# Selection and validation of a set of reliable reference genes for quantitative *sod *gene expression analysis in *C. elegans*

**DOI:** 10.1186/1471-2199-9-9

**Published:** 2008-01-22

**Authors:** David Hoogewijs, Koen Houthoofd, Filip Matthijssens, Jo Vandesompele, Jacques R Vanfleteren

**Affiliations:** 1 Department of Biology and Center for Molecular Phylogeny and Evolution, Ghent University, B-9000 Ghent, Belgium; 2 Center for Medical Genetics Ghent, Ghent University Hospital, B-9000 Ghent, Belgium

## Abstract

**Background:**

In the nematode *Caenorhabditis elegans *the conserved Ins/IGF-1 signaling pathway regulates many biological processes including life span, stress response, dauer diapause and metabolism. Detection of differentially expressed genes may contribute to a better understanding of the mechanism by which the Ins/IGF-1 signaling pathway regulates these processes. Appropriate normalization is an essential prerequisite for obtaining accurate and reproducible quantification of gene expression levels. The aim of this study was to establish a reliable set of reference genes for gene expression analysis in *C. elegans*.

**Results:**

Real-time quantitative PCR was used to evaluate the expression stability of 12 candidate reference genes (*act-1*, *ama-1*, *cdc-42*, *csq-1*, *eif-3.C*, *mdh-1*, *gpd-2*, *pmp-3*, *tba-1*, *Y45F10D.4*, *rgs-6 and unc-16*) in wild-type, three Ins/IGF-1 pathway mutants, dauers and L3 stage larvae. After geNorm analysis, *cdc-42*, *pmp-3 *and *Y45F10D.4 *showed the most stable expression pattern and were used to normalize 5 *sod *expression levels. Significant differences in mRNA levels were observed for *sod-1 *and *sod-3 *in *daf-2 *relative to wild-type animals, whereas in dauers *sod-1*, *sod-3*, *sod-4 *and *sod-5 *are differentially expressed relative to third stage larvae.

**Conclusion:**

Our findings emphasize the importance of accurate normalization using stably expressed reference genes. The methodology used in this study is generally applicable to reliably quantify gene expression levels in the nematode *C. elegans *using quantitative PCR.

## Background

Real-time quantitative PCR (qPCR) has become a very powerful tool for gene expression studies. One of the main difficulties associated with this highly sensitive technique is the necessity of accurate normalization, to account for varying amounts of cDNA input. This variation is inherent to the multistep process required to extract and process the RNA. The use of internal controls or reference genes has become the method of choice to account for this source of variation. The choice of an appropriate internal standard is therefore critical for relative gene expression analysis in order to obtain consistent and reliable results, especially when measuring small expression differences. A suitable reference gene to which expression can be normalized should have constant expression in all samples under investigation and should be insensitive to varying experimental treatments.

Although the nematode *C. elegans *is a commonly used model organism that has proven its importance in the unraveling of many important signaling pathways, to date no comprehensive analysis has been performed to validate candidate reference genes for gene expression analysis. Therefore, commonly used reference genes such as *act-1 *and *ama-1 *are often used without validating their usefulness. However, several reports indicate that the expression of commonly used reference genes can vary under different experimental conditions [1-4], possibly leading to dramatic misinterpretation of the expression level of a target gene. Although there is no universally accepted approach for data normalization, the method of using multiple stably expressed reference genes is currently the golden standard [5]. The straightforward method developed by Vandesompele and colleagues [6] to identify the most stably expressed reference genes from a set of candidate control genes can be used to normalize gene expression levels (geNorm). Their method also allows the determination of the optimal number of genes required for reliable normalization of qPCR generated gene expression data. They advocate use of the geometric mean of multiple stably expressed reference genes for normalization of relative quantities. This approach has been widely implemented by many researchers and has been statistically validated by Szabo et al. [7] and by the bootstrap procedure of Gabrielsson et al. [8], but surprisingly seems to be neglected in the *C. elegans *research field.

The Ins/IGF-1 signaling (IIS) pathway is a well-known life span regulator in *C. elegans*, *Drosophila *and mice [9,10]. A reduced activity of the pathway in *C. elegans *leads to nuclear localization of the transcription factor DAF-16, causing dauer formation and extended adult life span. Long-lived IIS mutants are highly resistant to a wide diversity of stressors, including enhanced survival upon exposure to the superoxide generator paraquat [11]. Given the potential role of reactive oxygen species in the ageing process, it is assumed that enzymes involved in the breakdown of ROS play an important role in the longevity phenotype of dauers and long-lived IIS mutants. Five different genes encoding superoxide dismutase (SOD) have been predicted in *C. elegans*. *sod-1 *and *sod-5 *encode cytosolic CuZnSODs. *sod-4 *expresses two splice variants, one membrane bound and one secreted. *sod-2 *and *sod-3 *encode mitochondrial MnSODs. It is well-established that increased life span is often associated with increased stress resistance and high antioxidant activity. For example, the long life span of dauers and IIS mutants is associated with increased stress resistance and high SOD activity [12-15]. Northern blot and microarray analysis have shown that *sod-3 *and possibly also *sod-5 *are upregulated in dauers and *daf-2 *mutants [16-18].

We demonstrate the usefulness of geNorm to determine the expression levels of the *sod *genes in *C. elegans*. geNorm analysis evaluates the stability of candidate reference genes based on the mean pairwise variation of a gene with all other tested genes. We compared the expression level of the candidate reference genes in 6 different *C. elegans *samples to validate internal controls for the analysis of *sod *gene expression. The presented approach can be applied to accurately normalize the expression of any *C. elegans *gene of interest.

## Results

We carefully selected 12 potential reference genes from different functional classes to minimize the chance of coregulation using two different approaches (Table 1). First we selected commonly used reference genes such as *ama-1 *(RNA polymerase II), *act-1 *(actin), *eif-3.C *(Translation initiation factor), *gpd-2 *(Glyceraldehyde-3-phosphate dehydrogenase) and *tba-1 *(tubulin). In a second approach we screened publicly available *C. elegans *microarray expression data [19,20]. In this procedure we ranked all genes according to their standard deviation in 553 experiments and chose potential reference genes within the top ranked ones based on the following three criteria: (1) functional description available, (2) expression data available for at least 500 experiments and (3) cDNA confirmed (to exclude erroneously predicted genes). This strategy resulted in *cdc-42*, *pmp-3*, *rgs-6*, *unc-16 *and *Y45F10D.4 *as candidate reference genes. After initial screening, we found that two genes (*rgs-6 *and *unc-16*) from the available expression data in Kim et al. [19] were expressed at very low levels and therefore excluded for further analysis.

**Table 1:** Function of candidate reference genes. Amplification efficiency is determined using the formula 10^-1/slope^. For the actual calculations, the base of the exponential amplification function is used (e.g. 1.96 means 96% amplification efficiency).

**Gene symbol**	**Sequence name**	**Function**	**Primerconc. (nM)**	**Amplification efficiency**
*act-1*	T04C12.6	Cytoskeletal structural protein	200	1.96
*ama-1*	F36A4.7	RNA polymerase II	400	1.85
*cdc-42*	R07G3.1	RHO GTPase	200	1.95
*csq-1*	F40E10.3	Calcium ion binding protein	200	1.95
*eif-3.C*	T23D8.4	Translation initiation factor	300	1.92
*mdh-1*	F20H11.3	Malate dehydrogenase	300	1.93
*gpd-2*	K10B3.8	Glyceraldehyde-3-phosphate dehydrogenase	300	1.94
*pmp-3*	C54G10.3	Acyl-CoA transporter	300	1.89
*tba-1*	F26E4.8	Alpha tubulin	200	1.95
*Y45F10D.4*	Y45F10D.4	Iron binding protein involved in Fe-S cluster formation	200	2.07

In an attempt to validate the remaining 10 candidate reference genes as internal control for analysis of *sod *gene expression in *C. elegans*, we set up an experiment containing young adult wild-type (N2), three strains carrying mutations in the Ins/IGF pathway (*daf-2*, *daf-16 and daf-2; daf-16*), wild type dauer worms and wild-type L3 stage larvae. Data were collected using RNA from 3 replicate *C. elegans *cultures.

Since the reverse transcription step is the source of most of the variability in quantitative PCR [21], cDNA synthesis was performed in duplicate from each of the three different biological replicates and pooled to use as template in the PCR experiments. For every primer pair concentrations were optimized in a range between 200 nM and 400 nM, based on a standard curve made with dilution series of a mixture of all cDNA templates. These standard curves produced efficiencies between 1.85 and 2.07 (Table 1) with standard deviations ranging from 0.019 to 0.065 and correlation coefficients greater than 0.984, as determined by qBase [22]. Three identical real-time qPCR experiments were performed. In each experiment, the expression levels of the candidate reference genes and *sod *genes were measured in duplicate in 6 different worm samples. The candidate control genes display a wide expression range with mean cycle threshold (Ct) values between 14.6 (*act-1*) and 28.1 (*ama-1*).

**Figure 1:**
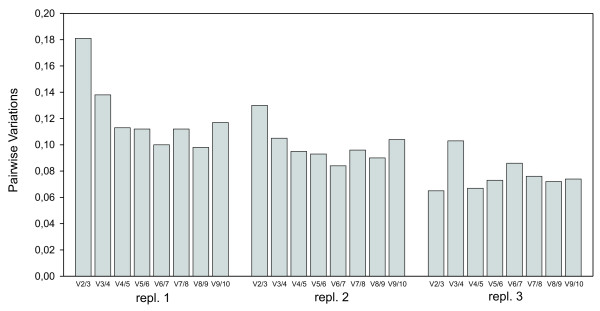
**Pairwise variations (*V*_*n/n*+1_) for all three biological replicate experiments**. A large variation between two sequential normalisation factors means that the added gene has a significant effect and should preferably be included for calculation of the normalisation factor. Addition of a 4^th ^reference gene makes no further improvement of the normalisation factor in each of the three replicate assays.

To identify the most stable reference genes we applied the geNorm program. The ranking of the candidate reference genes according to their stability measure value (M) in every biological replicate is displayed in Table 2. This gene expression stability measure M is the mean pairwise variation between a candidate reference gene and all other tested candidates. A higher value of M means greater variation in expression. The stepwise elimination of genes with the highest M values allows the ranking of the tested genes according to their expression stability. Interestingly, in the three biological repeat experiments *cdc-42 *and *pmp-3 *turned out to be among the three best performing reference genes. Moreover, in each of the three biological repeats the ranking of the least stable genes included *mdh-1*, *act-1 *and *gpd-2*. The consistent ranking of the best and worst reference genes illustrates the excellent correspondence and reproducibility between the 3 independently grown nematode cultures. Pairwise variations V_n/n+1 _between each combination of sequential normalization factors (NF) were calculated by geNorm to determine the optimal number of reference genes required for accurate normalization. Based on the recommendations by Vandesompele et al. [6] we used a cut-off value of 0.15, below which the inclusion of an additional reference gene does not result in a significant improvement of normalization (Figure 1). Following this criterion the inclusion of a third reference gene is required for assay set 1, but not for analyzing replicate sets 2 and 3 (V_2/3 _values 0.130 and 0.065, respectively). For consistency reasons we prefer to use the 3 most stably expressed genes for all 3 replicate assays (as also suggested as a general rule of thumb in Vandesompele et al. [6]).

**Table 2:** Ranking of candidate reference genes in order of their expression stability, decreasing from top to bottom. Average expression stability values (M) are shown between brackets. The 2 most stable genes cannot be ranked in order because of the required use of gene ratios for gene stability.

**Replicate 1**	**Replicate 2**	**Replicate 3**
*pmp-3*/*cdc-42 (0.117)*	*cdc-42/Y45F10D.4 (0.323)*	*Y45F10D.4/pmp-3 (0.092)*
*csq-1 (0.412)*	*pmp-3 (0.387)*	*cdc-42 (0.164)*
*Y45F10D.4 (0.502)*	*tba-1 (0.429)*	*tba-1 (0.293)*
*tba-1 (0.562)*	*csq-1 (0.470)*	*csq-1 (0.328)*
*eif-3.C (0.625)*	*eif-3.C (0.522)*	*ama-1 (0.375)*
*ama-1 (0.671)*	*gpd-2 (0.559)*	*eif-3.C (0.450)*
*act-1 (0.747)*	*ama-1 (0.627)*	*act-1 (0.502)*
*gpd-2 (0.799)*	*act-1 (0.688)*	*gpd-2 (0.551)*
*mdh-1 (0.889)*	*mdh-1 (0.770)*	*mdh-1 (0.601)*

**Figure 2:**
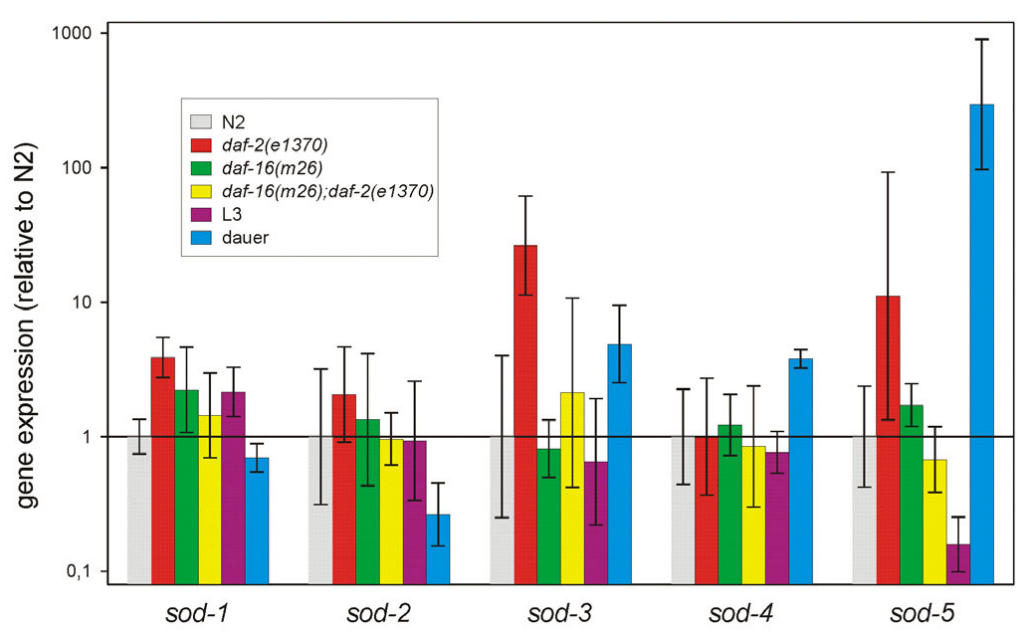
**Normalized *sod *mRNA expression levels**. The relative expression ratios are the average values from 3 replicate cultures. Bars indicate the 95% confidence interval of the mean (non-overlapping intervals denote significant differences at the 0.05 level). This statistic is a conservative criterion of statistical significance. All *sod *expression levels were normalized using the geometric mean of *cdc-42*, *pmp-3 *and Y45F10D.4.

To demonstrate the need for accurate relative quantification using suitable reference genes, the expression of the *sod *genes was studied. In each of the three assays a normalization factor based on the geometric mean of *cdc-42*, *pmp-3 *and Y45F10D.4 expression level was used to determine the relative expression level of all 5 *sod *genes. The average normalised *sod *mRNA values and the 95% confidence intervals are shown in Figure 2. The 95% confidence intervals clearly illustrate that *sod-1 *and *sod-3 *are significantly upregulated in *daf-2 *relative to N2, in agreement with microarray studies of McElwee et al. [18] and Murphy et al. [17]. In dauers, *sod-1 *is significantly downregulated relative to L3, *sod-3 *is significantly upregulated relative to L3, and *sod-4 *and *sod-5 *are significantly upregulated relative to both L3 and N2.

We illustrate that normalization to a single reference gene without appropriate validation can lead to erroneous results by normalizing *sod-1 *to *act-1*, *gpd-2 *and *mdh-1*, respectively. When normalized only to *act-1 *the expression level of *sod-1 *in *daf-2 *differs by more than 40 % and is not found to be significantly upregulated. Moreover the expression level of *sod-1 *in the dauer is upregulated instead of downregulated relative to L3. This shows that *act-1 *is unsuitable as a reference gene. When using *mdh-1*, only a modest, but not significant, increase by 2-fold in *daf-2 *could be seen, and the expression level of *sod-1 *appears to be strongly upregulated in dauers. Moreover, if *gpd-2 *or *ama-1 *had been used, no significant differential expression would have been detected in *daf-2 *mutants or dauers.

## Discussion

An ideal reference gene is expressed at the same level in all cell types and under all experimental conditions. Numerous publications demonstrate that no single gene is able to fulfill these criteria. To reliably measure small expression differences, normalization using (ideally multiple) stably expressed reference genes is of crucial importance. This forced us to undertake a comprehensive analysis of different potential reference genes in 6 different *C. elegans *samples, including distinct developmental stages of the wild-type and adults of Ins/IGF pathway mutants.

We here provide validated assays for a newly developed set of multiple candidate reference genes.

The geNorm analysis revealed that *cdc-42 *and *pmp-3 *are among the 3 best performing reference genes identified in each of 3 biological replicates. They were picked up from a microarray screen of differentially regulated genes (the Kim database) underscoring the reliability of this strategy to identify new candidate reference genes. They are far more stably expressed than conventional reference genes, at least in this study. Since in all 3 biological repeats addition of a fourth reference gene did not substantially improve the newly calculated normalization factor, there was no need to include more reference genes. Therefore we recommend to use three reference genes since we generally found that this number allows reliable and accurate quantification with a minimal effort.

Our analysis showed that *mdh-1 *is the least stable reference gene, which is in concordance with a previously published paper of McElwee et al. [23] who demonstrated by microarray analysis that *mdh-1 *was downregulated in dauers. Similarly, Mendenhall et al. [24] have recently shown that *gpd-2 *was upregulated in *daf-2 *worms. Both studies suggest that *gpd-2 *and *mdh-1 *are downstream genes of DAF-16. As expected, geNorm ranked these genes as the two least stable reference genes in two of the three biological repeats. Interestingly, the most commonly used reference gene *act-1 *is one of the least stable reference genes as indicated by the geNorm analysis.

We used the reference genes to reveal the expression levels of *sod*-genes in long-lived Ins/IGF mutants and dauers. Our results largely confirm and extend previous studies using Norhern blots, SAGE and microarray analysis. The increased expression of *sod-3 *in dauers and *daf-2 *mutants has been well documented [16-18], [25-27]. Elevated expression of *sod-4 *in dauers was reported by Jones et al. [25]. We also detected an increased expression of *sod-1 *in *daf-2 *mutants which is in accordance with the increased activity of cytosolic SOD in long-lived Ins/IGF-1 pathway mutants [28] given that SOD-1 appears to be the most active CuZnSOD [29] (Matthijssens, unpublished data).

## Conclusion

This study demonstrates the importance of appropriate validation of internal reference genes chosen for gene expression analysis using real-time qPCR. We show that candidate reference genes for accurate normalization of gene expression levels in *C. elegans *can be identified from publicly available microarray databases. The methodology used in the present study enables accurate analysis of differential gene expression of any set of candidate genes.

## Methods

### Nematode culturing

In this experiment, we compared the expression levels of candidate reference genes and five *sod*-genes in dauers compared to L3 larvae, and in adults of the wild-type N2 strain and the mutant strains *daf-2(e1370ts)*, *daf-16(m26) *and *daf-2(e1370ts); daf-16(m26)*. Reduction-of-function mutation in the gene *daf-2*, which encodes the Ins/IGF-1 receptor, causes extension of life span. This effect requires intact activity of the FOXO transcription factor DAF-16 and is therefore suppressed in strains that carry reduction- or loss-of-function mutations in *daf-16*. Synchronous populations were initiated from eggs prepared by alkaline hypochlorite treatment of gravid adults. Worms were grown on cholesterol supplemented Nutrient Agar (OXOID) plates containing a lawn of freshly grown *E. coli *K12 cells [30]. Wild-type L3 worms were harvested after 30 hours of growth at 24°C. At harvest, worms were rinsed off the plates, washed with S-buffer (43.55 mM KH_2_PO_4_, 6.45 mM K_2_HPO_4 _and 100 mM NaCl in distilled water, pH 6), flash frozen and stored at -75°C until use. Since *daf*-*2 *(*e1370ts*) is a constitutive dauer former at 24°C, the cultures grown for harvesting adult worms of all strains were incubated at 17°C and shifted to 24°C after the animals had molted to the fourth larval stage. Samples were taken two days later. Dauers from N2 were obtained as described in Houthoofd et al. [14]. Briefly, agar plates (pH 7.0) were seeded with freshly prepared eggs, hemoglobin and autoclaved *E. coli *cells and incubated at 24°C. These conditions induce almost 100% dauer formation. Plates containing less than 99% dauers were discarded. Three independently grown replicates of all worm cultures were used to account for experimental variation.

### Isolation of RNA and cDNA synthesis

Total RNA was isolated using the RNeasy Midi Kit (Qiagen) according to manufacturer's instructions. All samples were DNase treated (Zymo Research). A NanoDrop ND 1000 spectrophotometer was employed to analyze RNA concentration and purity. First strand cDNA was synthesized from 2 μg RNA using an oligo(dT) primer and a Moloney murine leukemia virus reverse transcriptase (Fermentas) at 42°C for 1 h.

### Real-time RT-PCR

Full-length gene sequences were extracted from WormBase (Release WS170) and primers were designed by the Primer3 software [31] and tested for specifity using NCBI BLAST. The targets amplified by the primer pairs were evaluated with MFOLD software [32] in order to check for the formation of secondary structures at the site of primer binding. MFOLD analysis was performed using default settings and 50 mM Na^+^, 3 mM Mg^2+ ^and a temperature of 60°C (which is the annealing temperature of the primers). Primers were purchased from Invitrogen. Primer and amplicon information are listed in Table 3.

**Table 3:** Primer sequences for candidate normalization genes.

**Gene symbol**	**Sequence name**	**Forward primer**	**Reverse primer**	**Amplicon size**
*act-1*	T04C12.6	gctggacgtgatcttactgattacc	gtagcagagcttctccttgatgtc	114
*ama-1*	F36A4.7	cctacgatgtatcgaggcaaa	cctccctccggtgtaataatg	139
*cdc-42*	R07G3.1	ctgctggacaggaagattacg	ctcggacattctcgaatgaag	111
*csq-1*	F40E10.3	aactgaggttctgaccgagaag	tactggtcaagctctgagtcgtc	111
*eif-3.C*	T23D8.4	gctgagactgttaagggaatgg	gagcgaaacagtggcataaac	99
*mdh-1*	F20H11.3	ctcgtgacgatctcttcaacac	gtcatagacaccagccttcttgag	161
*gpd-2*	K10B3.8	ctccatcgactacatggtctacttg	agctgggtctcttgagttgtagac	151
*pmp-3*	C54G10.3	gttcccgtgttcatcactcat	acaccgtcgagaagctgtaga	115
*tba-1*	F26E4.8	gtacactccactgatctctgctgacaag	ctctgtacaagaggcaaacagccatg	194
Y45F10D.4	Y45F10D.4	gtcgcttcaaatcagttcagc	gttcttgtcaagtgatccgaca	139

Quantitative RT-PCR was carried out using a Rotor-Gene 2000 centrifugal real-time cycler (Corbett Research) using the Platinum SYBR Green qPCR SuperMix-UDG (Invitrogen). Each reaction contained: 12.5 μl of the Platinum SYBR Green qPCR SuperMix-UDG, 200 nM, 300 nM or 400 nM of forward and reverse primers and 5 μl cDNA (1:40 RNA dilution), to a final volume of 25 μl. Amplification was performed in 0.1 ml real-time PCR tubes (Corbett Research) placed in the 72-well rotor of the Rotor-Gene instrument. The cycling conditions were as follows: 50°C for 2 min, initial denaturation at 95°C for 2 min, followed by 45 cycles of 15 s at 95°C, 30 s at 60°C, and 30 s at 72°C (gain set at 8 for SYBR Green). Following the final cycle, melting curve analysis was performed to examine the specificity in each reaction tube (absence of primer dimers and other nonspecific products). The Rotor-Gene software allows automatic melting curve analysis for all tested samples in a given run. SYBR Green fluorescence of the generated products was continuously monitored throughout the temperature ramp from 60 to 99°C. The temperature rose in 1° increments with a 5 s hold at each degree. A single melt peak for each reaction confirmed the identity of each PCR product. Each assay included a no-template control for every primer pair. In addition, aliquots of each reaction mixture were analyzed by agarose gel electrophoresis to evaluate amplification of nonspecific products.

### Quantification and data analysis

The threshold cycle (Ct) values of the Rotor-Gene software version 6.0 (Corbett Research) were exported to qBase version 1.3.5, a free program for the management and automated analysis of qPCR data [22], for further analysis. All measurements were produced in duplicate, and for each primer set, reaction efficiency estimates were derived from standard curves that were generated using serial dilutions of a cDNA pool of all nematode samples. These were then used by qBase to transform the Ct values to relative quantities for analysis with geNorm 3.4 software [6]. The geNorm VBA applet for Microsoft Excel determines the most stable reference genes from a set of genes in a given panel of cDNA samples. After normalization using the geometric mean of the three most stable reference genes the normalized *sod *expression ratios were standardized to minimize inter-experimental variation. To this purpose, the normalized expression levels were converted into logarithmic values, divided by their standard deviation and multiplied by the mean standard deviation of the 3 experiments to calculate the mean standardized expression per nematode sample and its 95% confidence interval. Finally, all values were linearized again using a power function, and plotted in a graph (Willems et al., manuscript in preparation). Differential gene expression was considered significant when the 95% confidence interval of the mean expression levels did not overlap (equivalent to P < 0.05).

## Authors' contributions

DH and KH conceived and designed all experiments, analyzed data and wrote the manuscript; DH and FM performed experiments; JV provided expert input in data analysis and critically revised the manuscript; JRV supervised the study. All authors read and approved the final manuscript.
